# Parent Preferences for Delaying Insulin Dependence in Children at Risk of Stage III Type 1 Diabetes

**DOI:** 10.1089/dia.2019.0444

**Published:** 2020-07-27

**Authors:** Rachael L. DiSantostefano, Jessie Sutphin, Joseph A. Hedrick, Kathleen Klein, Carol Mansfield

**Affiliations:** ^1^ Janssen Research & Development, LLC, Titusville, New Jersey.; ^2^ RTI Health Solutions, Research Triangle Park, North Carolina.; ^3^ Janssen Research & Development, LLC, Raritan, New Jersey.

**Keywords:** Discrete choice, Autoantibody screening, Interception treatment

## Abstract

***Background:*** Autoantibody screening in type 1 diabetes (T1D) may reduce the chances of potentially life-threatening diabetic ketoacidosis (DKA) at diagnosis by allowing individuals at risk of progression to more actively monitor for and/or manage progression to insulin dependence. We investigated parents' preferences for treatments to delay the onset of insulin dependence in children who are at high risk of developing Stage III T1D.

***Methods:*** A web-based survey (*n* = 1501) was administered to a stratified sample of parents (children <18 years) in the United States from an online panel. Parents were told to hypothetically assume that their youngest child would become insulin dependent within 6 months or 2 years and were offered a series of choices between no treatment and two hypothetical treatments that would delay insulin dependence. Random-parameters logit analysis and maximum acceptable risks were used to evaluate the relative importance of treatment benefits and risks.

***Results:*** Most parents chose at least one active treatment (2% always chose monitoring only). For parents of children without T1D (*n* = 901), delaying insulin dependence and reducing the risk of long-term health complications and serious infection were the most important treatment attributes. In addition, parents of children with T1D (*n* = 600) also valued reducing the risk of hospitalizations due to DKA.

***Conclusions:*** When told to assume their child would develop Stage III T1D, most parents considered active treatments to delay progression. For medicines under development to delay insulin dependence in T1D, the preferences expressed in this survey provide guidance on acceptable benefit–risk trade-offs.

## Introduction

Type 1 diabetes is an autoimmune disease, an attack by the immune system on insulin-producing islet cells. The metabolic consequence of type 1 diabetes (T1D) is a severe reduction of endogenous insulin capacity, leading to the inability to control blood glucose levels. The disease most frequently occurs in those bearing high-risk human leukocyte antigen alleles, and the initiation of the autoimmune pathology can be detected, often years before symptomatic disease becomes evident, by the presence in the blood of two or more autoantibodies to pancreatic islet proteins such as insulin, zinc transporter 8, and glutamic acid decarboxylase. Nearly all individuals who are positive for two or more autoantibodies will eventually require insulin. A staging system for T1D based on the presence of autoantibodies has been proposed, with Stage I characterized by the presence of ≥2 autoantibodies, Stage II characterized by ≥2 autoantibodies plus impaired glucose tolerance, and Stage III characterized as symptomatic diabetes, at which point exogenous insulin use is typically required.^1^ Because presymptomatic disease is difficult to detect without blood tests, most affected individuals progress to Stage III disease unaware of their condition and then present to the emergency department with very high blood glucose values and, quite often, in a state of diabetic ketoacidosis (DKA).^2–4^ Beyond the immediate health care costs of DKA, the presence of DKA at T1D onset is associated with an increased risk of hospitalization and negative long-term outcomes, including poor glycemic control and adverse neurocognitive impacts.^5–7^ Therefore, preventing DKA at T1D diagnosis may provide long-term value.^8^

Population-based screening programs for autoimmune conditions, including T1D, are a subject of increasing importance to identify patients at high risk of T1D.^1,9–12^ The primary benefit of T1D screening is avoiding DKA at diagnosis and its complications, which can be life threatening, and associated costs.^13^ An additional benefit of screening is that individuals and families have time to learn about treatment options for T1D and can be prepared when the disease does progress to Stage III.

Such screening programs, if made more widely available, could also significantly accelerate the development of therapies to “intercept” symptomatic disease in Stage I or Stage II and either delay the onset of Stage III T1D or, ideally, halt progression altogether. The recently reported results of the Teplizumab Prevention Trial, in which interception treatment with teplizumab resulted in a median delay of 24 months, are a clear indication that this is indeed an achievable goal.^14^ Such interception treatments offer a potential proactive alternative to passive monitoring by parents of children who are progressing. As these treatments are developed and evaluated, it will be important to understand the preferences of individuals at risk of developing T1D and their parents regarding the potential benefits of delaying disease onset and the level of risk and side effects they might be willing to accept. The American Diabetes Association standards of care emphasize the important role of patient preferences and values in clinical decision-making.^15^ Thus, the objective of this study was to investigate parents' preferences for treatments to delay the onset of insulin dependence in children who will develop Stage III T1D, including parents of children who have T1D and parents of children who do not.

## Methods

To elicit parents' preferences for T1D interception treatments for children, we designed and conducted a discrete-choice experiment (DCE) survey in accordance with good research practices.^16^ The DCE method assumes that respondents' treatment choices between hypothetical multiattribute treatments depend on the relative importance of the levels of the included attributes. Statistical analysis reveals the implicit importance of weights of the attributes and levels, consistent with observed patterns of choices.

The study was reviewed and granted an exemption from full review by the RTI International Institutional Review Board and complied with the Declaration of Helsinki.

### Survey development

**FIG. 1.**
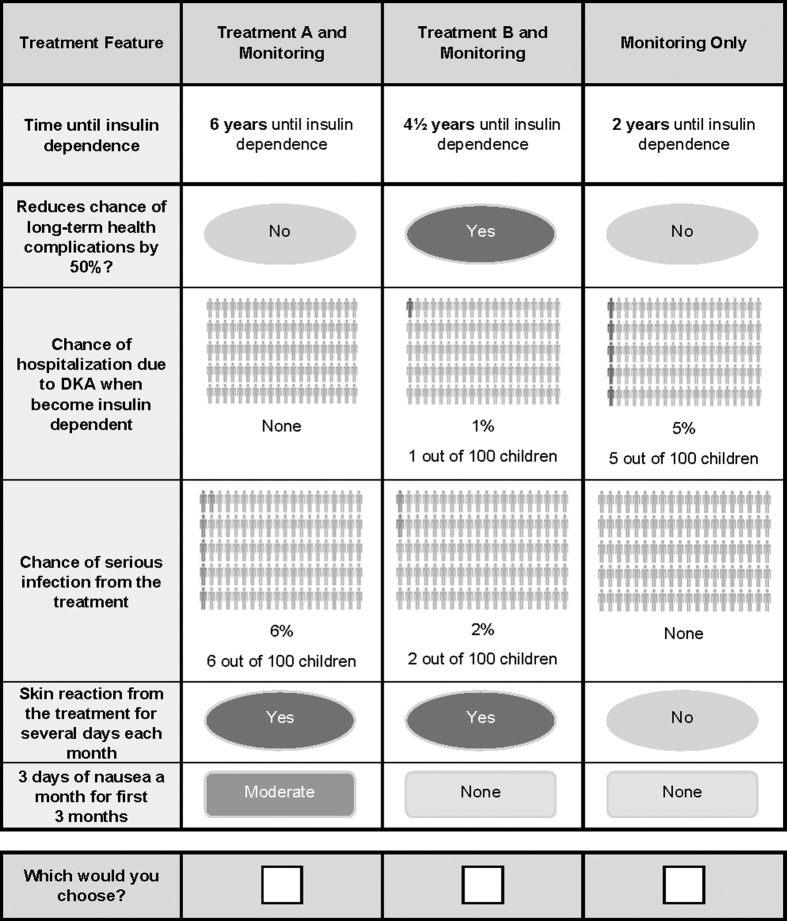
Example DCE question: Which treatment option would you choose for your child? The choice task shown here is one example of a DCE choice question from the survey. An experimental design determined the combination of attributes and levels for each hypothetical treatment and the pairs of hypothetical treatments shown in each DCE question. The experimental design included a total of 72 DCE questions, which were used to create nine blocks of eight DCE questions each. Respondents were randomly assigned to one of the nine blocks of eight questions. The hypothetical interception treatments presented in each DCE question were defined by a set of attributes, each with a set number of levels over which the attribute varied, whereas the monitoring-only option was fixed across the study design (Table 1). DCE, discrete-choice experiment.

In an online survey, parents were told to imagine the doctor gave their child a routine screening test for T1D and that the test was positive. Their child will develop T1D and become dependent on insulin. Parents were randomly assigned to a baseline time until insulin dependence of 6 months or 2 years. Each parent was presented with a series of DCE questions presenting two hypothetical treatments, defined by six attributes with varying levels (Table 1), that delayed insulin dependence and the option of monitoring only after screening. Figure 1 presents an example DCE question.

**Table 1.** Attributes and Levels

Attribute	Levels for experimentally designed treatments with monitoring	Levels for monitoring only
Time until insulin dependence^a^	4 Additional years	No additional time^b^
	2.5 Additional years	
	6 Additional months	
Reduces chance of long-term health complications by 50%^c^	Yes	No
	No	
Chance of hospitalization due to DKA when child becomes insulin dependent	None	5% (5 out of 100 children)
	1% (1 out of 100 children)	
	4% (4 out of 100 children)	
	5% (5 out of 100 children)	
Chance of serious infection from the treatment^d^	None	None
	2% (2 out of 100 children)	
	6% (6 out of 100 children)	
Skin reaction from the treatment for several days each month	No	No
	Yes	
3 Days of nausea a month for first 3 months^e^	None	None
	Mild	
	Moderate	

^a^ Respondents were shown their time until insulin dependence at baseline (6 months or 2 years until insulin dependence without treatment) plus additional years.

^b^ For monitoring only, respondents were shown either 2 years until insulin dependence or 6 months until insulin dependence depending on the respondent's baseline assignment.

^c^ Long-term complications include vision problems, kidney disease, heart disease, and nerve damage.

^d^ Serious infections include diseases such as pneumonia, tuberculosis, or a fungal infection. Serious infections can be treated, although your child may need to go to the hospital. In rare cases, serious infections can be life threatening if left untreated.

^e^ Nausea levels: none (no nausea), mild (may feel queasy, but it will not impact their appetite), and moderate (might feel the urge to vomit and will not feel like eating).

DKA, diabetic ketoacidosis.

Before finalizing the survey, 10 semistructured face-to-face qualitative interviews with parents of children with T1D (*n* = 4) and parents who do not have children with T1D (*n* = 6) were conducted to gain insight into the potential benefits and risks or concerns associated with an interception treatment. In addition, a focus group of parents of children with T1D was recruited through a local chapter of JDRF, a global charitable organization funding T1D research (*n* = 5), to review descriptions of T1D and its management included in the draft survey and to provide additional insight on the possible benefits and concerns associated with treatment to delay the onset of insulin dependence. The treatment attributes defining the DCE choice options were selected based on the results from the parent interviews, the characteristics of emerging treatments, and review by clinical experts with experience treating T1D. The range of levels for the attributes was selected to span the clinically relevant range of outcomes, improvements in effectiveness, and adverse events that might be expected based on some candidate therapies. In addition to the DCE questions, the survey also collected information about respondents' demographic and disease-experience characteristics. The survey instrument was also pretested in 15 semistructured, face-to-face interviews of parents with a child with T1D and parents who did not have a child with T1D and was refined based on input from the interviews before it was administered to the study sample.

In the final survey, the combinations of attribute levels for the two hypothetical treatments presented in each DCE question were created by an experimental design that varied the attribute levels independently to support analysis of the relative importance of each attribute level. The design was created in Sawtooth Software using a D-efficient algorithm to construct a fractional factorial experimental design.^17–21^ The design was evaluated and judged to have sufficient attribute-level balance and low correlation among levels. The experimental design included 72 DCE questions, split into nine blocks of eight questions each, and respondents were randomly assigned to one of the nine blocks. Outside of the experimental design, a ninth question with dominated no treatment and treatment options was added as a means to evaluate patient comprehension and attendance to the survey. One treatment option had both more favorable efficacy and safety relative to the other no treatment and treatment alternatives.

### Study population

Dynata, a health care market research company, invited potentially eligible respondents from a set of online panels, including panels where individuals self-identified as having T1D or having a family member with T1D to participate in the survey. Eligible respondents were parents of a child aged 2–17 years, were aged 18 years or older, were U.S. residents, and were able to read and understand English and provide informed consent. The sample was stratified based on the child's age (ages 2–6, 7–10, and 11–17 years) and whether or not the respondent's child had T1D. Potential respondents received an e-mail invitation to the survey. After answering the screening questions and providing informed consent, eligible respondents completed the survey.

### Data analysis

The DCE data were analyzed using random-parameters logit to generate a relative preference weight for each attribute level.^22^ The difference between the most- and least-preferred levels of an attribute is the conditional relative importance of an attribute relative to the other attributes and range of levels in the study. The conditional relative importance for an attribute is calculated as the difference between the preference weights for the most- and least-preferred levels for that attribute. The results of the random-parameters logit also were used to calculate the maximum acceptable risk (MAR) of serious infection for an additional 2-year delay in time until insulin dependence and for a reduction in the chance of long-term health complications by 50%.

The sample of parents of children with T1D and the sample of parents whose children do not have T1D were analyzed separately, resulting in relative importance numeric values that could not be directly compared across the groups. In addition, subgroup analyses explored whether preferences varied systematically between mutually exclusive subgroups defined by child age, parent age, and parent gender.

## Results

### Sample

The final sample included 1501 parents: 901 with a child without T1D and 600 with a child with T1D. The respondents' demographic characteristics and their experience with T1D are presented in Supplementary Tables S1, S2, S3, S4. Among the respondents who completed the survey who did not have a child with T1D, 79% were married and 51% were employed full-time. The average (standard deviation [SD]) age of respondents was 42 (9.7) years, and 70% of the sample was female. Among the respondents who completed the survey and had a child with T1D, 78% of respondents were married and 72% were employed full-time. The average (SD) age of respondents was 37 (8.4) years, and 56% of the sample was female.

### Respondent preferences

The choices from the DCE questions suggest the average parent in the sample preferred a treatment to delay insulin dependence and monitoring, over monitoring only, when asked to assume their child had been screened and was going to become insulin dependent in 6 months to 2 years without additional treatment. Across the eight choice questions, each with varying levels of the attributes evaluated (Table 1 and Fig. 1), 98% of parents in the overall sample chose a treatment at least once, and 58% of parents always chose a treatment (63% among parents without a child with T1D, and 49% among parents with at least one child with T1D), irrespective of the range of benefits and risks presented.

### Relative attribute importance

**FIG. 2.**
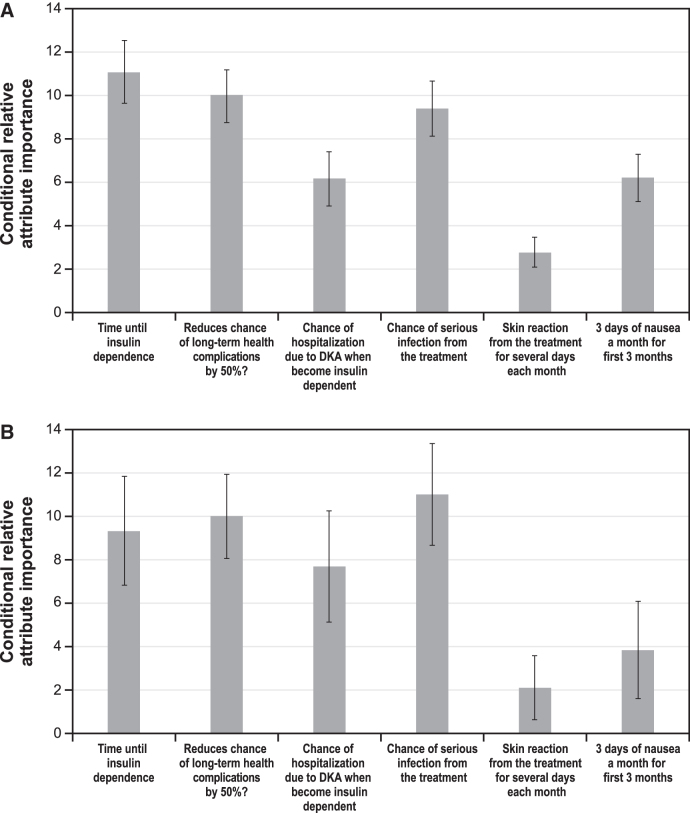

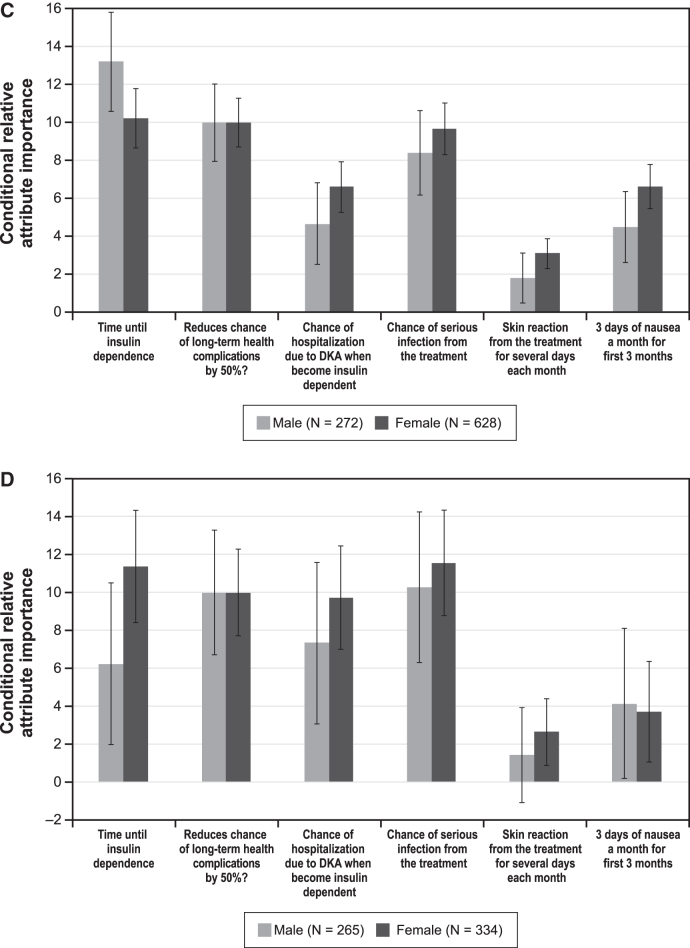
Conditional relative attribute importance. **(A)** Parents of children without T1D (*N* = 901). **(B)** Parents of children with T1D (*N* = 600). **(C)** Parents of children without T1D (*N* = 900*), by parent gender. **(D)** Parents of children with T1D (*N* = 599*), by parent gender. For ease of interpretation of relative importance, estimates from the random-parameters logit models were used to rescale attributes, where one attribute, 50% reduction in long-term complications, was rescaled to 10. The relative importance of each of the other attributes was scaled relative to the conditional importance of this attribute. Attributes with bar heights <10 had less relative importance over the ranges examined, and those with bar heights >10 had more relative importance over the ranges examined. The vertical bars surrounding each relative importance weight estimate denote the 95% confidence interval. Separate random-parameters logit models were used for the analyses of preferences of parents of children without and with T1D. *One respondent did not provide gender and was excluded from the model. DKA, diabetic ketoacidosis; T1D, type 1 diabetes.

Figure 2 shows the conditional relative attribute importance of changing each attribute from the least-preferred level to the most-preferred level among parents of children without T1D (Fig. 2A) and parents of children with T1D (Fig. 2B).

#### For parents of children without T1D

Among parents of children without T1D (*n* = 901), over the ranges presented in the survey, an increase in the additional time until insulin dependence from 6 months to 4 years, reducing the risk of long-term health complications, and avoiding a 6% risk of serious infection from the treatment were the most important attribute changes. Avoiding a 5% chance of hospitalization due to DKA and moderate nausea were approximately equally important. Avoiding skin reactions from the treatment was relatively less important compared with the ranges of the other attributes presented in the survey (Fig. 2A). Supplementary Data shows the normalized mean preference weight estimates for each attribute level for parents of children without T1D (Supplementary Fig. S1).

In subgroup analyses among parents of children without T1D, women's preferences were found to be statistically significantly different from men's preferences (*P* = 0.00055 for the joint significance of the interaction terms; see Supplementary Fig. S2 in the Supplementary Data for the normalized mean preference weight estimates). Generally, men placed more relative importance on delaying insulin dependence than women. In addition, women placed more relative importance on the risks of DKA and serious infection relative to men. However, differences in relative importance (Fig. 2C) were not statistically significantly different for any attributes.

No statistically significant differences in preferences were observed among subgroups defined by child age or parent age.

#### For parents of children with T1D

Among parents of children with T1D (*n* = 600), over the ranges presented in the survey, avoiding a 6% risk of serious infection from the treatment, reducing the risk of long-term health complications, increasing the additional time until insulin dependence from 6 months to 4 years, and avoiding a 5% chance of hospitalization due to DKA were the most important attribute changes (Fig. 2D). These changes were equally important. Going from 3 days of moderate nausea a month to no nausea and avoiding skin reaction from the treatment were relatively less important compared with the ranges of other attributes presented in the survey. Supplementary Data show the normalized mean preference weight estimates for parents of children with T1D (Supplementary Fig. S3).

In subgroup analyses among parents of children with T1D, women's preferences were found to be statistically significantly different from men's preferences (*P* = 0.00057 for the joint significance of the interaction terms; see Supplementary Fig. S4 in the Supplementary Data for the normalized mean preference weight estimates).

The relative importance calculations (Fig. 2D) highlight some differences between men and women. Women placed about equal weight on the additional time until insulin dependence from 6 months to 4 years and avoiding a 6% risk of serious infection, followed by approximately equal weight on a reduced risk of long-term health complications and avoiding a 5% risk of hospitalization for DKA. Men placed the most importance on a reduced risk of long-term health complications and avoiding a 6% risk of serious infection, followed by avoiding a 5% risk of hospitalization for DKA, and then the additional time until insulin dependence from 6 months to 4 years. Women demonstrated a preference for avoiding skin reactions from the treatment, whereas men did not.

No statistically significant differences in preferences were observed among subgroups defined by child age or parent age.

### Maximum acceptable risk

**FIG. 3.**
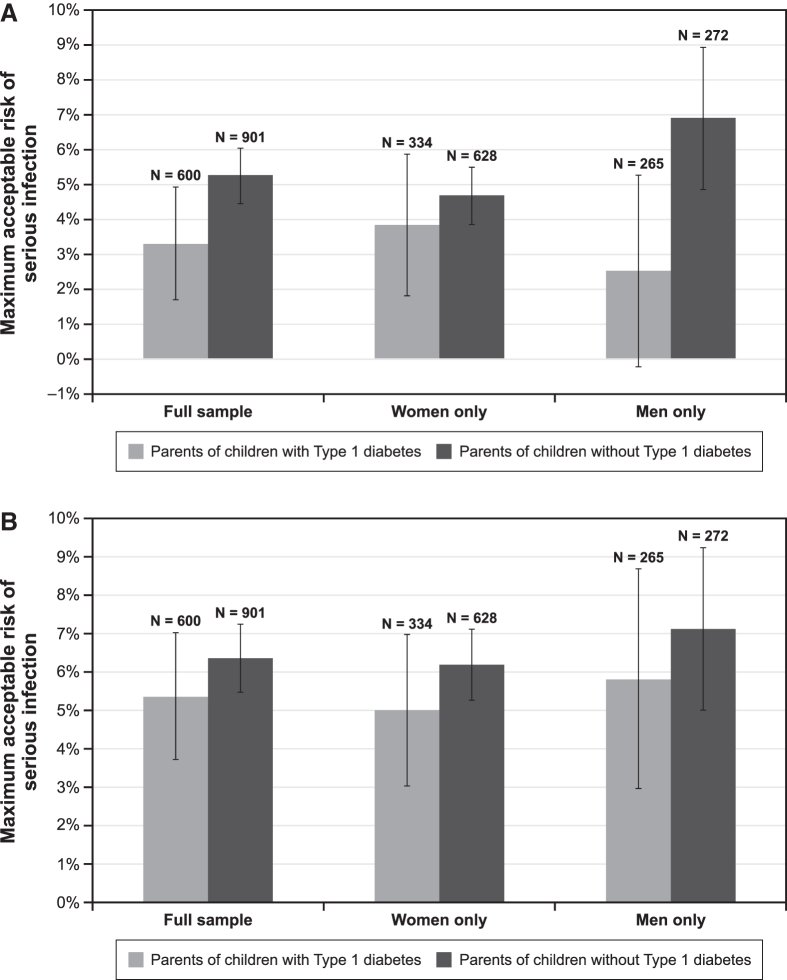
Maximum acceptable risk calculations. **(A)** Additional time until insulin dependence for 2 years delay of onset. **(B)** Fifty percent reduction in the chance of long-term health complications. Maximum acceptable risk estimates >6% are outside the range of the level of risk of serious infection presented in the survey and should be interpreted with caution. One respondent did not provide gender and was excluded from the model.

Figure 3 presents the MARs of serious infection for a treatment that improves time until insulin dependence by 2 years (Fig. 3A) and a treatment that reduces the chance of long-term health complications by 50% (Fig. 3B). The MAR of serious infection ranged from 2.5% to 6.9% for an additional 2-year delay in insulin dependence and from 5.0% to 7.1% for reductions in long-term health complications. Although results were not significantly different, parents of children without T1D were willing to accept higher risks, on average, of serious infection in exchange for 2 more years of insulin dependence and reducing long-term health complications relative to parents of children with T1D. There were no differences by parent gender.

## Conclusions

T1D is an autoimmune disease that imposes significant health, financial, and quality-of-life burdens on patients and their families. The merits of population-based screening for presymptomatic T1D are being studied in several countries, including the United States.^1,9–12^ With the advent of accurate screening tests that can identify children who are progressing to the insulin-dependent stage of T1D, a treatment to delay the onset of insulin dependence, if associated with better glucose control and reduced incidence of DKA, would provide benefits to patients and their families. Treatments to delay T1D progression are on the horizon, with some evidence that early intervention may prolong pancreatic function in newly diagnosed T1D^23,24^ and may delay Stage III T1D among those at Stage II.^14^

This study evaluated the parent perspective on the benefit–risk trade-offs in treating versus not treating Stage II T1D. This sample included parents with children <18 years of age and included both parents with at least one child with T1D and parents who did not have a child with T1D. Both groups were included, as experience with the disease is likely to affect benefit–risk trade-offs. For parents of children without T1D, delaying insulin dependence, reducing risk of long-term health complications, and the risk of serious infection were the most important treatment attributes, on average, in a hypothetical medicine over the ranges that we examined. These were also the three most important for parents of children with T1D in addition to reducing the risk of hospitalizations due to DKA.

These results are clinically important in two ways. First, the results suggest that parents in our sample are interested in treatments that may delay T1D progression, with the majority of participants choosing treatment each time irrespective of treatment benefits and risks. Less than 3% of respondents always selected monitoring only over a treatment in the DCE choice questions. Second, this study lends clarity to what risks would be acceptable to parents of children at risk for T1D and in exchange for what level of benefit, particularly knowing that with advance warning they could plan and most likely avoid the most serious complications at diagnosis (very high blood glucose and DKA). Parents in our study valued a treatment that would provide more time until insulin dependence, lower the risk of long-term health consequences from T1D, and reduce the risk of DKA at diagnosis. Although the risk of serious infection was a significant concern to parents, acceptable risk was within the known range of risks associated with existing immunotherapies (e.g., golimumab [Simponi]; Simponi prescribing information; Ref.^25^). This study explored the willingness of parents to accept the risk of side effects for the benefits from a treatment that would delay the onset of insulin dependence over a monitoring-only approach. The results will help researchers assess the extent of risk tolerance for the treatment and identify treatments that parents might find acceptable.

In the era of precision and personalized medicine, screening tests and preventive treatments are expected to become more common. Decisions around treatments for disease prevention can be complex, given the uncertainty around both the risk of getting the disease and the potential effectiveness of treatment. For T1D, the presence of two or more autoimmune antibodies means that patients eventually will develop insulin-dependent T1D; however, for other diseases, the screening test may only predict the risk of developing a disease. In all cases, understanding the perspectives of patients and caregivers for screening and preventive treatments is important for determining a potential disease management strategy. Studies on preferences for preventive and risk-reducing treatments have been conducted, for example, in individuals at risk of rheumatoid arthritis,^26,27^ women with the *BRCA1/2* gene,^28^ and individuals at risk of Alzheimer's disease.^29^

The results of the DCE survey should be interpreted in the context of limitations related to the survey instrument and sample. The survey presents hypothetical scenarios to respondents. Decisions made in the survey may not fully predict decisions made in a clinical setting where other considerations may come into play. The samples were convenience samples recruited through opt-in panels of individuals who self-reported T1D status in their children and who chose to participate in research. These individuals may not be representative of the broader population of parents of children with and without T1D. Our samples had higher rates of education, private insurance, and marriage than might be expected from the general population. In addition, the sample of parents who had a child with T1D contained a disproportionate number of parents who had T1D (27%) based on what would be expected given the prevalence of the disease. A significant fraction of the parents without a child with T1D reported having heard of DKA (46%) and being a caregiver of someone with T1D (19%). Therefore, benefit–risk trade-offs in this study and the proportion of parents that may accept treatment in the general population may be different than that seen in our sample.

In our study, the proportion of parents who would select treatment may be biased upward due to a labeling effect, in which there is recoding between “doing something” (treatment) and “doing nothing” (monitoring only).^30–32^ Finally, medical reimbursement of screening and treatment may affect the number of parents who might seek treatment. Nevertheless, the study demonstrates that there are parents who would choose to delay T1D in their child who is at increased risk based on their autoantibodies.

The results shown in this study are the preferences in delaying T1D for the average parent in the sample. However, parents' preferences for treatments to delay T1D insulin dependence are likely heterogeneous. Exploration of heterogeneity will need to be explored in future study. Among both samples of parents, including those who had children with T1D and those who did not, males and females had statistically significantly different preferences. On average, male parents of children without T1D prioritized additional time until insulin dependence over the risks, whereas male parents of children with T1D prioritized avoiding risks. On average, female parents (of both groups) prioritized both additional time until insulin dependence and avoiding risks. The other subgroups tested based on the child's age and the parent's age were not significantly different.

In conclusion, when told to assume their child would develop symptomatic (Stage III) T1D, on average, parents preferred a treatment to only monitoring progression, even understanding that such monitoring would likely reduce the risk of DKA at diagnosis (as compared with the current standard of care). Only a small fraction of the sample selected monitoring only in every treatment choice question. This held true for both parents who had a child with Stage III T1D as well as for parents with no affected children. Parent's preferences varied by gender, but the overall preference for a treatment to delay time until insulin dependence was consistent. The preferences expressed over the benefits and harms in this survey provide guidance on acceptable benefit–risk trade-offs for future treatments to delay insulin dependence in T1D. Understanding patients' and caregivers' willingness to undergo preventive therapy based on their perceptions of screening, risk of disease diagnosis, and treatment-related benefits and risks will be important in implementing screening and preventive strategies for T1D.

## Supplementary Material

Supplemental data

Supplemental data

Supplemental data

Supplemental data

## Assistance

Kimberly Moon of RTI Health Solutions provided overall project management for this study. Kate Lothman of RTI Health Solutions provided medical writing services, which were funded by Janssen R&D, LLC.
