# Supplementary material for: Parent Preferences for Delaying Insulin Dependence in Children at Risk of Stage III Type 1 Diabetes
Source: Diabetes Technol Ther. 2020 Jul 27;22(8):584–93. doi: 10.1089/dia.2019.0444 (PMC7406995; doi:10.1089/dia.2019.0444)
Supplement: Supplemental data [file Supp_TableS1.pdf]

Supplementary Table S1. Demographic Characteristics of Parents

Question	*Parents of children with T1D* (N=*600*)	*Parents of children without T1D* (N=*901*)	*Full sample* (N=*1501*)
How old are you?			
Mean (SD)	37.4 (8.4)	42.4 (9.7)	40.4 (9.5)
Median	36.0	41.0	39.0
Min, max	20, 66	18, 75	18, 75
What is your gender?, *n* (%)			
Female	334 (55.7)	628 (69.7)	962 (64.1)
Male	265 (44.2)	272 (30.2)	537 (35.8)
Other	0	0	0
Prefer not to answer	1 (0.2)	1 (0.1)	2 (0.1)
What is your marital status?, *n* (%)			
Single/never married	82 (13.7)	78 (8.7)	160 (10.7)
Married/living as married/civil partnership	470 (78.3)	707 (78.5)	1177 (78.4)
Divorced or separated	43 (7.2)	94 (10.4)	137 (9.1)
Widowed/surviving partner	4 (0.7)	21 (2.3)	25 (1.7)
Other	1 (0.2)	1 (0.1)	2 (0.1)
What type of health insurance do you have? (Please check all that apply), *n* (%)			
I do not have health insurance	10 (1.7)	40 (4.4)	50 (3.3)
Private insurance that I pay for myself	171 (28.5)	143 (15.9)	314 (20.9)
Private insurance that my or spouse's employer pays all or part of	288 (48.0)	510 (56.6)	798 (53.2)
Medicaid	93 (15.5)	136 (15.1)	229 (15.3)
Medicare	70 (11.7)	77 (8.5)	147 (9.8)
Veterans' Health Insurance	12 (2.0)	9 (1.0)	21 (1.4)
Other	13 (2.2)	20 (2.2)	33 (2.2)
Do not know or not sure	5 (0.8)	4 (0.4)	9 (0.6)
What is the highest level of education you have completed?, *n* (%)			
Less than high school	0	2 (0.2)	2 (0.1)
Some high school	2 (0.3)	14 (1.6)	16 (1.1)
High school or equivalent (e.g., GED)	68 (11.3)	114 (12.7)	182 (12.1)
Some college but no degree	83 (13.8)	160 (17.8)	243 (16.2)
Technical school	18 (3.0)	52 (5.8)	70 (4.7)
Associate's degree (2-year college degree)	72 (12.0)	98 (10.9)	170 (11.3)
4-year college degree (e.g., BA, BS)	228 (38.0)	275 (30.5)	503 (33.5)
Some graduate school but no degree	11 (1.8)	29 (3.2)	40 (2.7)
Graduate or professional degree (e.g., MBA, MS, MD, PhD)	118 (19.7)	157 (17.4)	275 (18.3)
Which of the following best describes your employment status?, *n* (%)			
Employed full-time	429 (71.5)	463 (51.4)	892 (59.4)
Employed part-time	60 (10.0)	92 (10.2)	152 (10.1)
Self-employed	29 (4.8)	46 (5.1)	75 (5.0)
Homemaker	52 (8.7)	202 (22.4)	254 (16.9)
Student	5 (0.8)	10 (1.1)	15 (1.0)
Retired	5 (0.8)	31 (3.4)	36 (2.4)
Disabled/unable to work	12 (2.0	27 (3.0)	39 (2.6)
Unemployed and looking for work	7 (1.2)	25 (2.8)	32 (2.1)
Unemployed and not looking for work	1 (0.2)	5 (0.6)	6 (0.4)

max, maximum; min, minimum; SD, standard deviation; T1D, type 1 diabetes.
