# Supplementary material for: Parent Preferences for Delaying Insulin Dependence in Children at Risk of Stage III Type 1 Diabetes
Source: Diabetes Technol Ther. 2020 Jul 27;22(8):584–93. doi: 10.1089/dia.2019.0444 (PMC7406995; doi:10.1089/dia.2019.0444)
Supplement: Supplemental data [file Supp_TableS2.pdf]

Supplementary Table S2. Child's Demographic Characteristics and Parent's Experiences with Type 1 Diabetes Among Parents of Children with Type 1 Diabetes

Question	*Parents of children with T1D (N = 600)*
All respondents	
How many children with T1D (age 2–17) do you have?	
1	553 (92.2%)
2	33 (5.5%)
3	4 (0.7%)
4	10 (1.7%)
How old is your child who has T1D? If you have more than one child with T1D, please tell us the age of the youngest child with T1D who is between the ages of 2 and 17	
2–6 Years old	200 (33.3%)
7–10 Years old	200 (33.3%)
11–17 Years old	200 (33.3%)
How old was your child when diagnosed with T1D? (years)[a]	
Mean (SD)	5.6 (3.9)
Median	5.0
Min, max	0.25, 17
How old is your child now? (years)	
Mean (SD)	9.1 (4.3)
Median	9.0
Min, max	2, 17
When first diagnosed with T1D, was your child hospitalized overnight?	
Yes	460 (76.7%)
No	140 (23.3%)
Among respondents whose child was hospitalized overnight	
Was your child diagnosed with DKA when they were diagnosed with T1D?	
N	460
Yes	313 (68.0%)
No	96 (20.9%)
I am not sure	51 (11.1%)
All respondents	
Very low blood sugar can be a medical emergency. Has your child ever had any serious *low* blood sugar events that required going to the emergency room or hospital?, *n* (%)	
Yes	377 (62.8%)
No	213 (35.5%)
I am not sure	10 (1.7%)
Does your child *currently* use any of the following to treat their T1D? (Please check all that apply)[b], *n* (%)	
Insulin pump	345 (57.5%)
CGM	326 (54.3%)
Neither of these	85 (14.2%)
Among respondents whose child currently uses an insulin pump	
About how long has your child been using an insulin pump?	
N	345
<1 year	58 (16.8%)
1–2 Years	169 (49.0%)
>2 but <5 years	90 (26.1%)
>5 years	28 (8.1%)
I am not sure	0
Among respondents whose child currently uses a CGM	
About how long has your child been using a CGM?	
N	326
<1 year	73 (22.4%)
1–2 Years	151 (46.3%)
>2 but <5 years	83 (25.5%)
>5 years	19 (5.8%)
I am not sure	0
All respondents	
Has your child's doctor given them a target range for A1C?	
Yes	446 (74.3%)
No	89 (14.8%)
I am not sure	65 (10.8%)
Among respondents whose child's doctor gave them a target range for A1C	
How often does your child meet their A1C target?	
N	446
All or most of the time	192 (43.0%)
Some of the time	208 (46.6%)
Not very often	34 (7.6%)
Never	12 (2.7%)
I am not sure	0
All respondents	
Have any of your children ever had a screening test for T1D before they developed symptoms?	
Yes	260 (43.3%)
No	321 (53.5%)
I am not sure	19 (3.2%)

(*continued*)

Supplementary Table S2. (Continued)

[a]Answer choices ranged from 1 to 17 years and included two additional options, <6 months and 6 months to <1 year. Less than 6 months was assigned a value of 0.25 years, and 6 months to <1 year was assigned a value of 0.75 years.

[b]This question allows for multiple responses; therefore, the total number of responses may not add up to the total number of people who answered the question.

A1C, glycated hemoglobin; CGM, continuous glucose monitor; max, maximum; min, minimum.
