# Supplementary material for: Parent Preferences for Delaying Insulin Dependence in Children at Risk of Stage III Type 1 Diabetes
Source: Diabetes Technol Ther. 2020 Jul 27;22(8):584–93. doi: 10.1089/dia.2019.0444 (PMC7406995; doi:10.1089/dia.2019.0444)
Supplement: Supplemental data [file Supp_TableS3-S4.pdf]

Supplementary Table S3. Child's Demographic Characteristics and Parent's Experiences with Type 1 Diabetes Among Parents of Children Without Type 1 Diabetes

Question	*Parents of children without T1D* (N=*901*)
All respondents	
How old is your youngest child who is between the ages of 2 and 17? (By age stratification goal)	
2–6 Years old	301 (33.4%)
7–10 Years old	300 (33.3%)
11–17 Years old	300 (33.3%)
How old is your youngest child between the ages of 2 and 17? If you have more than one child between the ages of 2 and 17, please think about your youngest child when you answer the questions in this survey (years)	
Mean (SD)	8.8 (4.6)
Median	8.0
Min, max	2, 17
All respondents	
Have you ever known anyone with T1D?	
Yes	542 (60.2%)
No	299 (33.2%)
I am not sure	60 (6.7%)
Among respondents who have known anyone with T1D	
Have you ever been a caregiver for someone with T1D?	
n	542
Yes	103 (19.0%)
No	438 (80.8%)
I am not sure	1 (0.2%)
All respondents	
Before taking this survey, had you ever heard of DKA?	
Yes	410 (45.5%)
No	439 (48.7%)
I am not sure	52 (5.8%)
Have any of your children ever been tested by a doctor or other health care professional for T1D?	
Yes	178 (19.8%)
No	573 (63.6%)
I am not sure	150 (16.6%)

max, maximum; min, minimum.

Supplementary Table S4. Parent's Experiences with Type 1 Diabetes and Other Conditions

Question	*Parents of children with T1D* (N=*600*), n *(%)*	*Parents of children without T1D* (N=*901*), n *(%)*	*Full sample* (N=*1501*), n *(%)*
Do you have T1D?			
Yes	161 (26.8)	14 (1.6)	175 (11.7)
No	423 (70.5)	875 (97.1)	1298 (86.5)
I am not sure	16 (2.7)	12 (1.3)	28 (1.9)
Do any other adults living in your household have T1D?			
Yes	124 (20.7)	34 (3.8)	158 (10.5)
No	465 (77.5)	860 (95.4)	1325 (88.3)
I am not sure	11 (1.8)	7 (0.8)	18 (1.2)
Before taking this survey, were you aware that T1D can cause long-term health problems?			
Yes	548 (91.3)	730 (81.0)	1278 (85.1)
No	39 (6.5)	149 (16.5)	188 (12.5)
I am not sure	13 (2.2)	22 (2.4)	35 (2.3)
Has your child ever been hospitalized due to a serious infection?			
Yes	197 (32.8)	82 (9.1)	279 (18.6)
No	392 (65.3)	809 (89.8)	1201 (80.0)
I am not sure	11 (1.8)	10 (1.1)	21 (1.4)
Has your child ever experienced red, itchy, or tender skin from a shot, vaccination, or other type of injection?			
Yes	274 (45.7)	175 (19.4)	449 (29.9)
No	306 (51.0)	685 (76.0)	991 (66.0)
I am not sure	20 (3.3)	41 (4.6)	61 (4.1)
In the past year, has your child experienced moderate nausea as described in [Table 1]?			
Yes	345 (57.5)	265 (29.4)	610 (40.6)
No	236 (39.3)	610 (67.7)	846 (56.4)
I am not sure	19 (3.2)	26 (2.9)	45 (3.0)
