# Supplementary material for: Parent Preferences for Delaying Insulin Dependence in Children at Risk of Stage III Type 1 Diabetes
Source: Diabetes Technol Ther. 2020 Jul 27;22(8):584–93. doi: 10.1089/dia.2019.0444 (PMC7406995; doi:10.1089/dia.2019.0444)
Supplement: Supplemental data [file Supp_Data.pdf]

Supplementary Data

Preference Weights

Parents of children without type 1 diabetes

Supplementary Figure S1 shows the normalized mean preference weight estimates for each attribute level for parents of children without type 1 diabetes (T1D). The preference weights indicate the ranking of levels within each attribute (i.e., a higher preference weight indicates that a level is more preferred).

Preferences for attribute levels were ordered as expected, with better levels being preferred to worse levels. On average, parents wanted a treatment over monitoring only. The disutility of monitoring only, compared with a treatment option combined with monitoring, is represented by the large and negative parameter on the monitoring-only constant. Respondents preferred more time until insulin dependence, an interception treatment that reduces the chance of long-term health complications by 50%, no chance of hospitalization when their child becomes insulin dependent, no chance of serious infection from the treatment, no skin reaction from the treatment, and no nausea.

The levels within each attribute were statistically significantly different from one another with two exceptions. There

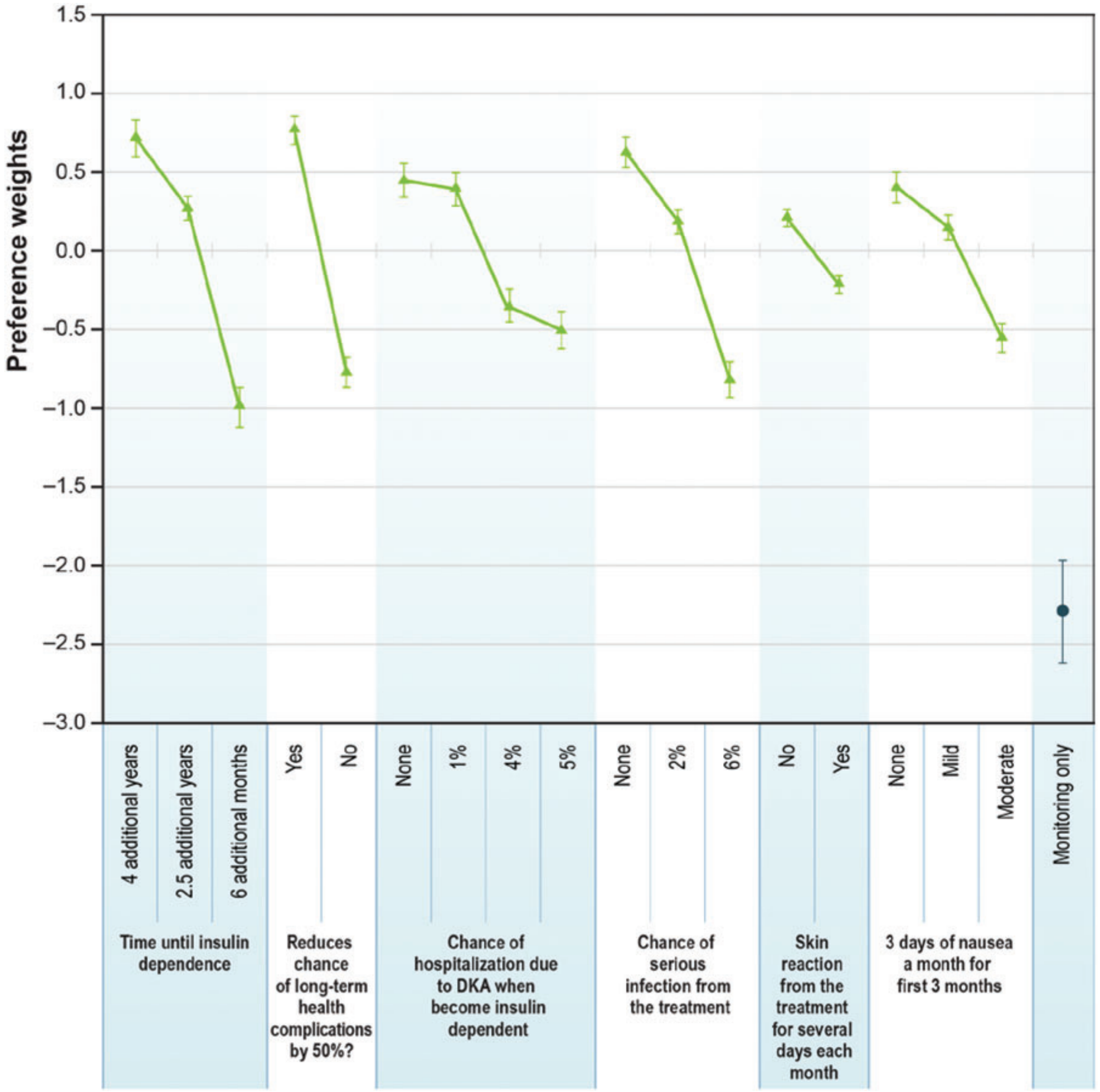

SUPPLEMENTARY FIG. S1. Preference weights for parents of children without type 1 diabetes ($N = 901$). The vertical bars surrounding each mean preference weight denote the 95% confidence interval. DKA, diabetic ketoacidosis.

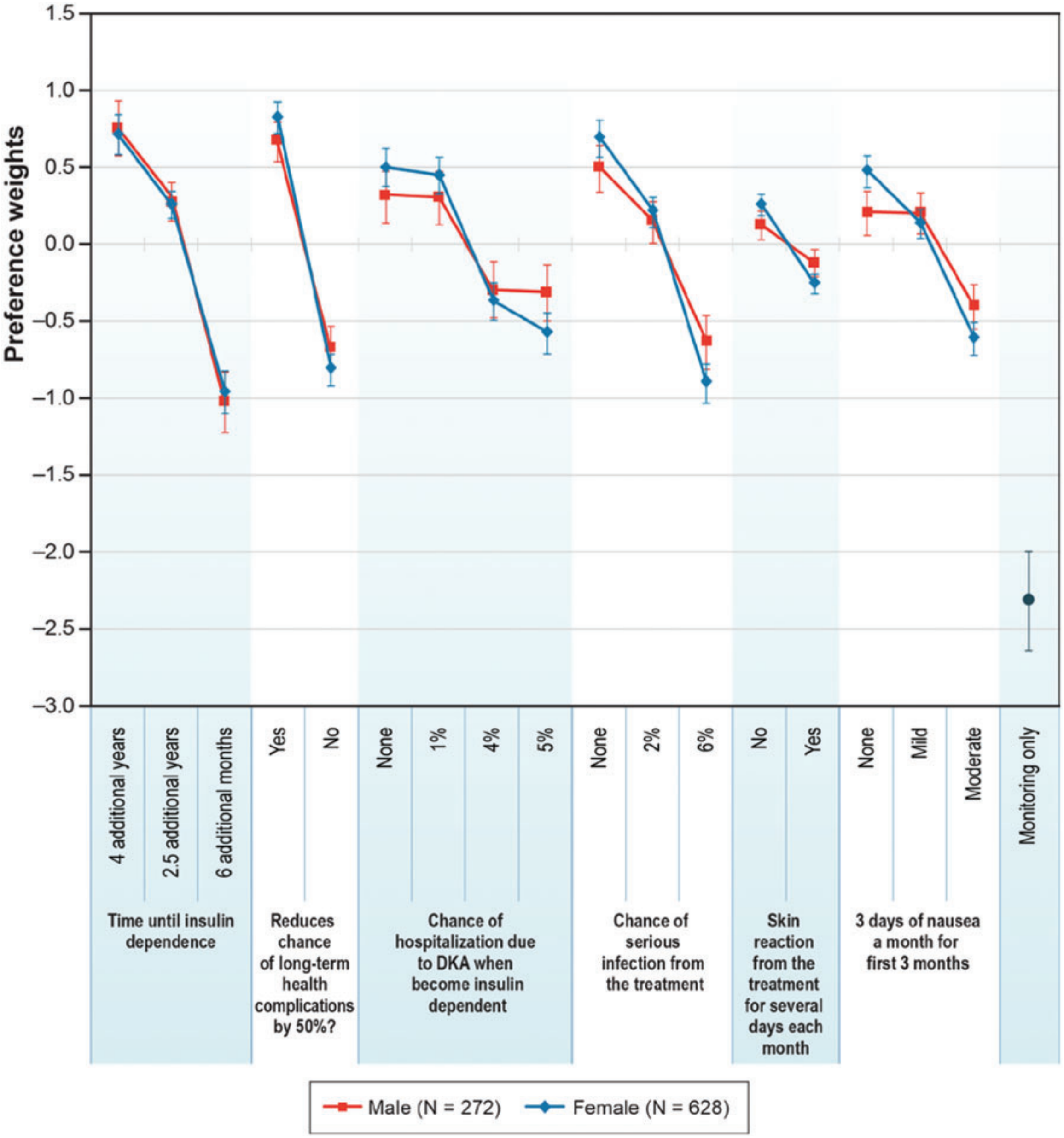

SUPPLEMENTARY FIG. S2. Subgroup preference weights by parent gender for parents of children without type 1 diabetes (N=900). The vertical bars surrounding each mean preference weight denote the 95% confidence interval. One respondent did not provide gender and was excluded from the model.

was no statistically significant difference between a 1% risk of hospitalization due to diabetic ketoacidosis (DKA) at diagnosis and a 0% risk. There was also no statistically significant difference between a 4% and 5% risk of hospitalization due to DKA at diagnosis for parents of children without T1D.

The scale of the preference weights is arbitrary, but differences between preference weights for an attribute can be interpreted as a measure of expected change in well-being associated with changes in attribute levels. Larger differences between preference weights indicate that respondents viewed the change as relatively more important. For example, a change in time until insulin dependence from 2.5 additional years to 4 additional years had a relative importance of ∼0.448 (preference weights, 0.272–0.720). Likewise, moving from a 5% chance of hospitalization due to DKA to a 0% chance had a value of ∼0.952 (preference weights, –0.500 to 0.452). Therefore, moving from 5% to a 0% chance of hospitalization was approximately two times more important than moving from 2.5 additional years to 4 additional years until insulin dependence.

Supplementary Figure S2 shows the normalized mean preference weight estimates for each attribute level for the parent gender subgroup model for parents of children without T1D. The test for the joint significance of the interaction terms for women's preferences found the interaction terms were

statistically significantly different from men's preferences ($P = 0.00055$ for the joint significance of the interaction terms).

Parents of children with T1D

Supplementary Figure S3 shows the normalized mean preference weight estimates for each attribute level for parents of children with T1D.

Preferences for attribute levels were ordered as expected, with better levels being preferred to worse levels. On average, respondents wanted a treatment over monitoring only. The disutility of monitoring only compared with a treatment option combined with monitoring is represented by the large and negative parameter on the monitoring-only constant. Respondents preferred more time until insulin dependence, an interception treatment that reduces the chance of long-term health complications by 50%, reduced risk of hospitalization when their child becomes insulin dependent, no chance of serious infection from the treatment, no skin reaction from the treatment, and no nausea.

Most of the levels within each attribute were statistically different from one another ($P < 0.05$). The exceptions were that there was no statistically significant difference between having 3 days of mild nausea and 3 days of moderate nausea a month. There was also no statistically significant difference between a 1% chance of hospitalization due to DKA at diagnosis and a 0% chance.

A change in time until insulin dependence from 2.5 additional years to 4 additional years had a relative importance of ~0.196 (preference weights, 0.095–0.291). Likewise, moving from a 5% chance of hospitalization due to DKA to a 0% chance has a value of ~0.557 (preference weights, –0.334 to 0.223). Therefore, moving from 5% to a 0% chance of hospitalization was nearly three times more important than

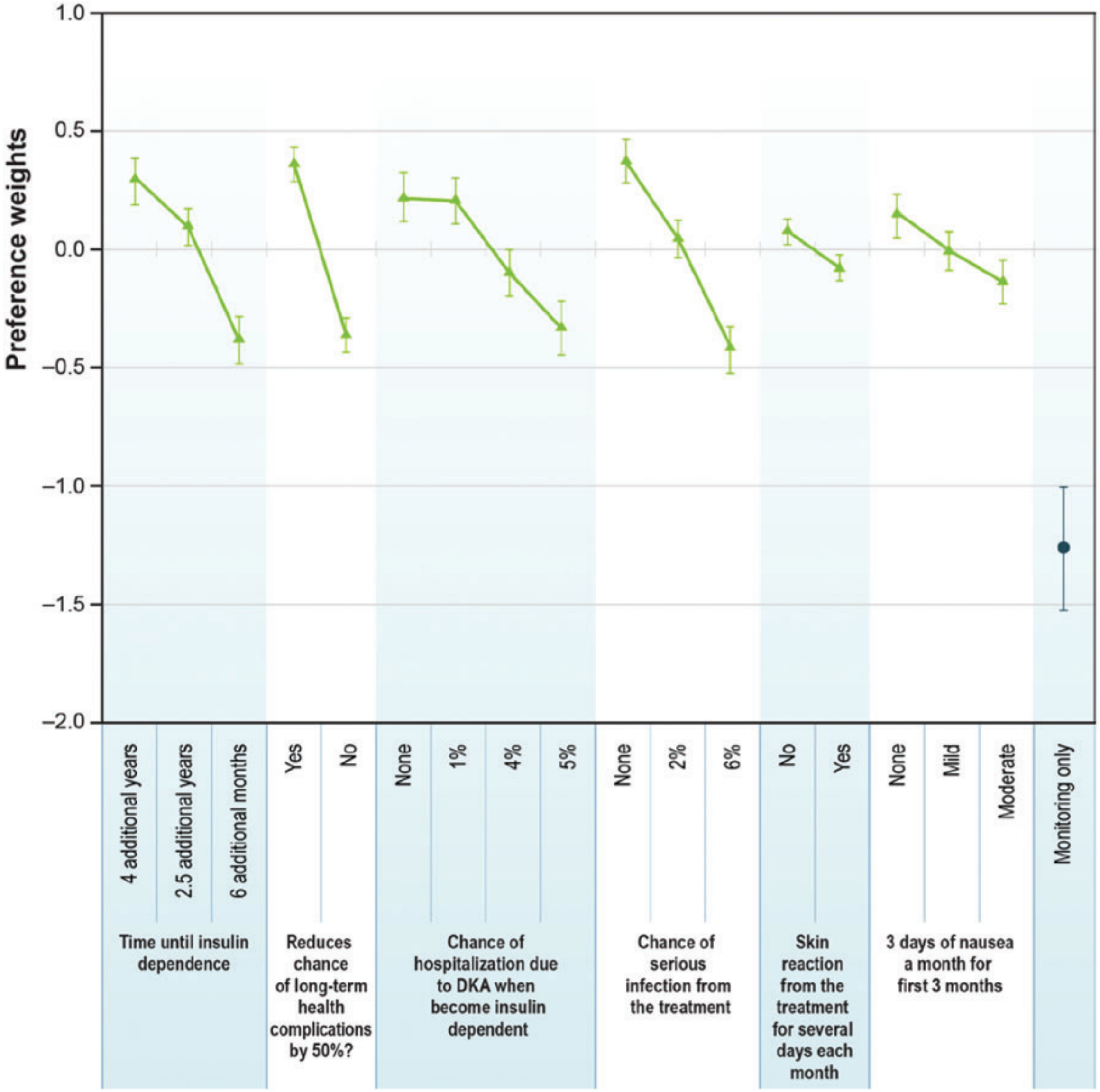

SUPPLEMENTARY FIG. S3. Preference weights for parents of children with type 1 diabetes ($N = 600$). The vertical bars surrounding each mean preference weight denote the 95% confidence interval.

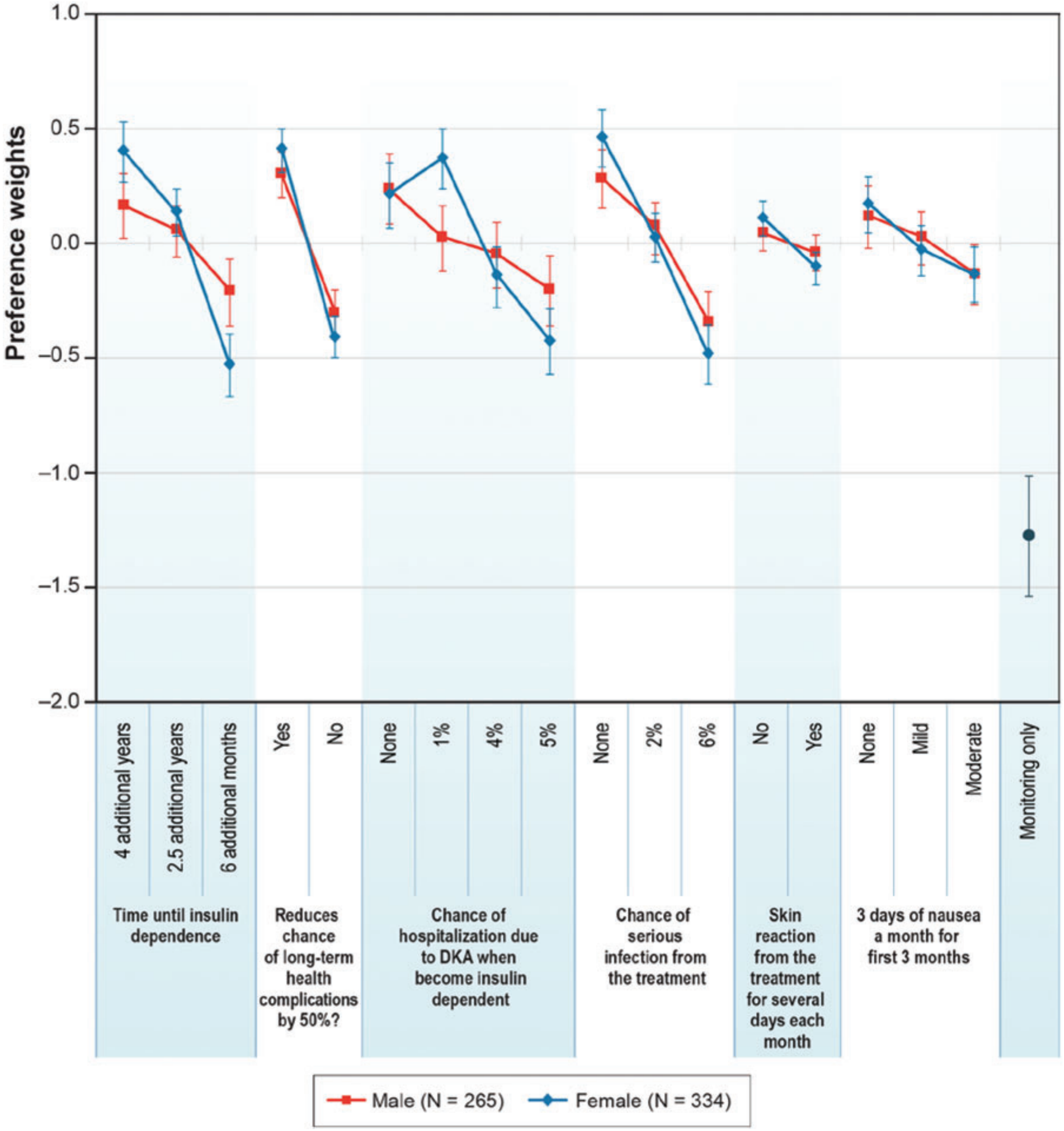

SUPPLEMENTARY FIG. S4. Subgroup preference weights by parent gender for parents of children with type 1 diabetes ($N = 599$). The vertical bars surrounding each mean preference weight denote the 95% confidence interval. One respondent did not provide gender and was excluded from the model.

moving from 2.5 additional years to 4 additional years until insulin dependence.

Supplementary Figure S4 shows the normalized mean preference weight estimates for each attribute level for the parent gender subgroup for parents of children with T1D. One respondent did not provide gender and was excluded from the model. The test for the joint significance of the interaction terms for women's preferences found that the interaction terms were statistically significantly different from men's preferences ($P = 0.00057$).
